# Parkinson's Disease-Related Risk of Suicide and Effect of Deep Brain Stimulation: Meta-Analysis

**DOI:** 10.1155/2020/8091963

**Published:** 2020-09-27

**Authors:** Juncong Du, Xi Liu, Xuan Zhou, Hui Wang, Wen Zhou, Jin Jiang, Wuxue Peng, Lijuan Mo, Changhong Tan, Lifen Chen

**Affiliations:** Department of Neurology, The Second Affiliated Hospital of Chongqing Medical University, 74 Linjiang Road, Chongqing, China

## Abstract

**Background:**

Previous studies investigated the risk of suicide in patients with Parkinson's disease (PD) but reported discrepant results. Deep brain stimulation (DBS) is an effective therapy for PD, while its effect on suicide risk has seldom been researched. This meta-analysis aimed to estimate the risk of suicide and/or suicidal ideation in PD patients and in PD patients who underwent DBS.

**Methods:**

Relevant articles published in the PubMed or EMBASE or CNKI database from 1990 to December 2019 were sourced, and the combined standardized mortality rate (SMR) or odds ratio (OR) was pooled.

**Result:**

A total of 1070 articles were found. After screening, 4 cross-sectional studies, 4 cohort studies, 2 randomized controlled trial studies, and 2 case-control studies were included in this meta-analysis. Pooled data indicated that PD patients may have increased risk of suicide (lnSMR, 0.459; 95% confidence interval (CI), 0.286 to 0.632; *p* < 0.001). No significant difference was found in the risk of suicide when comparing PD patients who underwent DBS with PD patients who received only drug therapy (OR = 2.844, 95%CI: 0.619 to 13.072, *p*=0.179). DBS may increase the risk of suicide and/or suicidal ideation in PD patients compared with general population (lnSMR = 3.383, 95%CI: 2.839 to 3.927, *p* < 0.001).

**Conclusion:**

PD patients have higher risk of suicide and/or suicidal ideation compared with controls, while PD patients who received DBS tend to have an increased risk of suicide or suicidal ideation. Psychological evaluation is needed in PD patients, and pre- and post-operation evaluations are necessary for PD patients who underwent DBS.

## 1. Introduction

Parkinson's disease (PD) is the second most common neurodegenerative disorder that affects 2–3% of the population ≥65 years of age [1]. It is reported that standardized incidence rates of PD are 8–18 per 100 000 person-years, affecting all races and ethnicities [2]. PD patients may experience various non-motor symptoms, including cognitive impairment, behavioral changes, somatosensory disturbances, and depression [3], among which depression is the most prevalent non-motor symptom in PD patients [4]. The most unacceptable result of depression is suicide. Many risk factors other than depression have also been identified contributing to suicide and/or suicidal ideation in PD patients, including non-motor symptoms, motor complications, and the increased disability [1], and our understanding of suicidality in PD patients is still limited [5]. The specific prevalence rate of suicide and/or suicidal ideation in PD patients varies between studies, and some studies even found that the prevalence of suicide in PD patients was lower than that in general population [6]. Considering the high prevalence of PD worldwide, the suicide and/or suicidal ideation in PD patients may affect a large population, and it is necessary to verify the prevalence of suicide and/or suicidal ideation in PD patients.

Deep brain stimulation (DBS) has emerged as one of the most effective treatments for movement disorders, especially when the effectiveness of drug therapy is limited or adverse effects of drug therapy such as dyskinesia exist [7]. However, as a relatively novel therapy, although the safety of DBS treatment is generally well demonstrated [8], its effect on suicide and/or suicidal ideation has seldom been investigated. Limited studies have shown that DBS treatment may increase the rate of suicide in PD patients [9–11]. Therefore, the risk of suicide in PD patients who underwent DBS treatment should be noticed in clinic, and it is helpful to verify the specific prevalence of suicide and/or suicidal ideation in PD patients who underwent DBS. Additionally, whether the different targeted nucleus for DBS treatment affects the rate of suicide and/or suicidal ideation in PD patients is still unknown.

To verify the prevalence of suicide and/or suicidal ideation in PD patients, and in PD patients who underwent DBS treatment, we pooled the prevalence of suicide and/or suicidal ideation in PD patients among different studies. We also tried to analyze the effect of different targeted nucleus on risk of suicide and/or suicidal ideation in PD patients who underwent DBS treatment, but there is insufficient study for meta-analysis.

## 2. Materials and Methods

### 2.1. Search Strategy

We searched literature published in the PubMed or EMBASE or CNKI database from 1990 to December 2019 in English or Chinese, using the following search strategy: ((((Parkinson disease) OR Parkinson's disease) OR Paralysis Agitans) OR Parkinsonism) AND Suicide.

### 2.2. Inclusion and Exclusion Criteria

Studies were initially screened by titles and abstracts by 2 investigators separately, and arguments between the 2 investigators were solved by a third investigator. The full text of the article was further evaluated after screening titles and abstracts. Inclusion criteria were as follows: (1) randomized controlled trials, cohort studies, case–control studies, or cross-sectional studies; (2) studies that compared patients with PD with patients without PD, or studies that compared PD patients who received DBS with PD patients did not receive DBS, or studies that compared PD patients who received DBS with controls (healthy population or general population); and (3) the outcomes of interest including suicide ideation, suicide attempts, or completed suicide, and the risk estimates including standardized mortality ratio (SMR), hazard ratio (HR), relative risk (RR), or odds ratio (OR).

Exclusion criteria were as follows: (1) review, case report, letter, commentary, and other nonarticle type literature; (2) no analysis on the discrete risk of PD on suicide ideation, suicide attempts, or completed suicide; and (3) studies with incomplete data or risk estimates could not be calculated.

### 2.3. Assessment of Quality of Literature

All studies were independently evaluated by 2 researchers for quality assessment. The Newcastle-Ottawa Quality Assessment Scale (NOS) [12] was used for evaluation of cohort and case–control studies. For cross-sectional studies, a modified Newcastle-Ottawa Scale [13] was used. The Cochrane Collaboration's Tool for Assessing Risk of Bias [14] was used for assessment of randomized controlled trials. Disagreements were discussed and resolved by discussing with a third researcher.

### 2.4. Data Selection

The following data were collected from each included article: the name of first author, publication year, country in which the study was performed (if multiple countries were involved in one single study, the continent or world was collected), study design, risk estimates, number of included individuals, diagnostic criteria of PD, evaluation method or criteria of suicide (including suicide ideation, suicide attempts, or completed suicide), and the specific brain nucleus (subthalamic nucleus (STN) or globus pallidus internus (GPi)) chosen for DBS.

### 2.5. Statistical Analysis

Data were expressed as mean/median (lower 95% confidence interval, upper 95% confidence interval). Studies reported SMR or OR as risk estimates were pooled separately. For combination of SMRs, lnSMRs were used for meta-analysis, while for ORs, OR values were used directly for pooling. Meta-analysis was performed using the Stata 12.0 software (StataCorp LP, College Station, USA) to estimate the risks of suicide in PD patients. *p* < 0.05  was considered statistically significant. Heterogeneity among studies was assessed using the *χ*^2^ test and *I*^2^ statistic. Heterogeneity was considered signiﬁcant when the *p*-value of the *χ*^2^ test <0.1 or *I*^2^ ≥ 50%. If signiﬁcant heterogeneity according to the Mantel–Haenszel analysis was identified, the random-effects model was used to pool data; otherwise, the fixed-effects model was used. Sensitivity analysis was performed to address the influence of every single study on the overall heterogeneity. The trim-and-fill method was used to estimate publication bias.

## 3. Results

### 3.1. Data Selection and Characteristics

**Figure 1:**
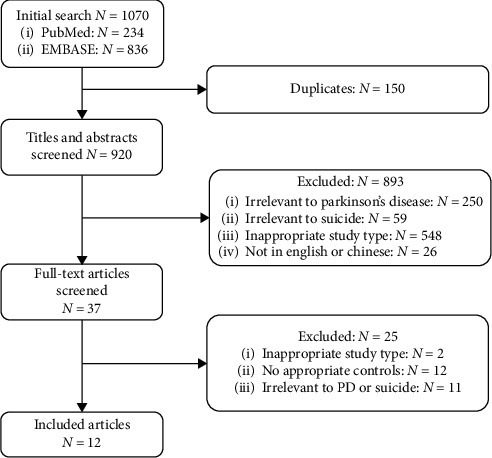
Flowchart of literature screening.

234 articles were obtained from PubMed, 836 articles were obtained from EMBASE, and no article was obtained from the CNKI database. 920 articles remained after exclusion of duplicates. 893 articles were excluded by screening the titles and abstracts, mainly, because of inappropriate study type (e.g., reviews, case reports, letters) or because irrelevant to PD or suicide. Among the remaining 37 articles, after reading the full text, 2 were excluded because of their inappropriate study type, 12 were excluded because of absence of appropriate controls, and 11 were excluded because they are irrelevant to PD or suicide. Finally, only 12 articles met the inclusion criteria. The detailed process of study selection is presented in a flow chart in Figure 1.

The included 12 articles consisted of 4 cross-sectional studies [9, 15–17], 4 cohort studies [18–21], 2 randomized controlled trial studies [10, 11], and 2 case–control studies [6, 22] (Supplementary Tables S1 and S2).

### 3.2. Quality of Literature

We evaluated each study using the NOS and The Cochrane Collaboration's Tool for Assessing Risk of Bias (Supplementary Tables S3–S6). Of 9 possible points, the median score for cohort studies was 7.25, and the score for 2 case–control studies was 6. Of 8 possible points, the median score for cross-sectional studies was 5. Of 6 possible points, the score for 2 RCT studies was 4 and 6.

### 3.3. PD Patients May Have an Increased Risk of Suicide

**Figure 2:**
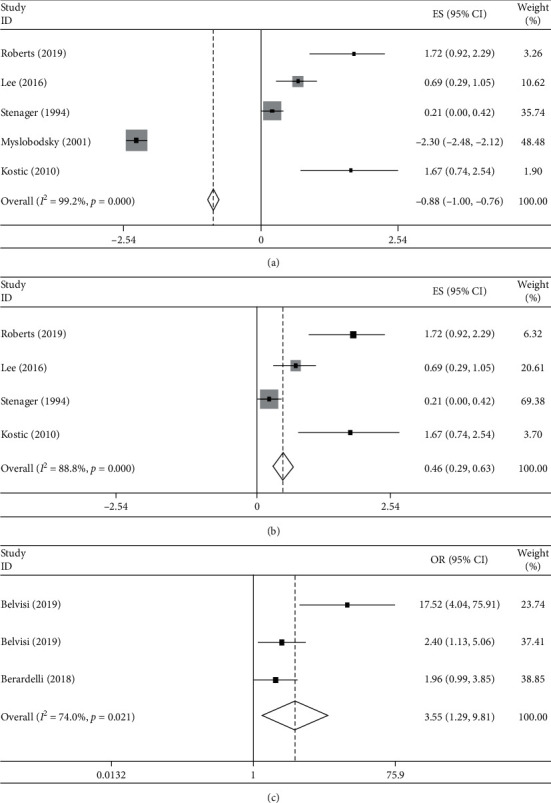
Forest plots of studies comparing Parkinson's disease (PD) patients to controls. (a) Forest plots of studies comparing PD patients with general population showed a decreased risk of suicide in PD patients. (b) After excluding Myslobodsky's study, forest plots of studies comparing PD patients with general population showed a significantly increased risk of suicide in PD patients. (c) Forest plots of studies comparing PD patients with non–central nervous system disease controls (healthy, psoriasis, or glaucoma) showed an increased risk of suicide in PD patients.

We pooled lnSMRs of 5 studies on risk of suicide in PD patients, the results presented a pooled lnSMR of −0.880 (95%CI: −1.004 to −0.755, *p* < 0.001) with significant heterogeneity (*I*^2^ = 99.2%, *p* < 0.001) (Figure 2).

We noted that one study [6] reported a completely opposite result compared with the other studies and had a high weight. This study reported a much higher suicide rate of general population compared with the other 4 studies (0.8% compared with 0.012%, 0.33%, 0.03%, and 0.37%, respectively), which may be attributed to the high suicide rate in USA population [23, 24]. Therefore, we performed another meta-analysis excluding this study [6]. After excluding this study, the pooled lnSMR became 0.459 (95%CI: 0.286 to 0.632, *p* < 0.001) with significant heterogeneity (I^2^ = 88.8%, *p* < 0.001) (Figure 2).

For studies comparing PD patients with healthy controls or patients with non–central nervous system diseases (psoriasis and glaucoma), we pooled the OR of these studies. The results found that PD patients had a higher risk of suicide than controls (pooled OR = 3.551, 95%CI: 1.285 to 9.810, *p*=0.015) with significant heterogeneity (*I*^2^ = 74.0%, *p*=0.021) (Figure 2).

PD patients who underwent DBS have an increased risk of suicide compared with PD patients without DBS or general population.

**Figure 3:**
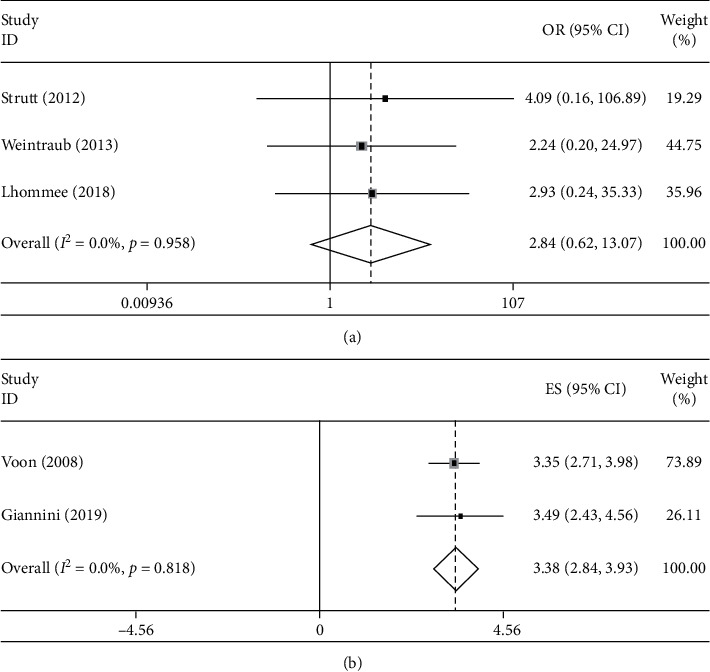
Forest plots of studies comparing Parkinson's disease (PD) patients who underwent deep brain stimulation (DBS) with controls. (a) Pooled data showed a trend of increase in risk of suicide in PD patients who underwent DBS compared with PD patients who received only drug therapy, but there is no statistical significance. (b) Pooled data showed PD patients who underwent DBS had an increased risk of suicide compared with general population.

For studies comparing PD patients who underwent DBS with PD patients who received only drug therapy, ORs of these studies were pooled. The results presented a potential trend of increased suicide risk in PD patients who underwent DBS (pooled OR = 2.844, 95% CI: 0.619 to 13.072, *p*=0.179) without heterogeneity (*I*^2^ < 0.01%, *p*=0.958), but there was no statistical significance. For the 2 studies comparing PD patients who underwent DBS with general population, lnSMRs of the 2 studies were pooled, and the results presented an increased risk of suicide in PD patients who underwent DBS (pooled lnSMR = 3.383, 95%CI: 2.839 to 3.927, *p* < 0.001) with almost no heterogeneity (*I*^2^ < 0.01%, *p*=0.818) (Figure 3).

There are not sufficient studies for analyzing the effect of different targeted nucleus on risk of suicide and/or suicidal ideation in PD patients who underwent DBS treatment. Only one study reported no significant difference in the rate of suicidal behavior and ideation between patients who received DBS in STN and GPi (1.5% vs. 0.7%, Fisher's exact test *p*=0.61). [10].

### 3.4. Sensitivity Analysis and Source of Heterogeneity

**Figure 4:**
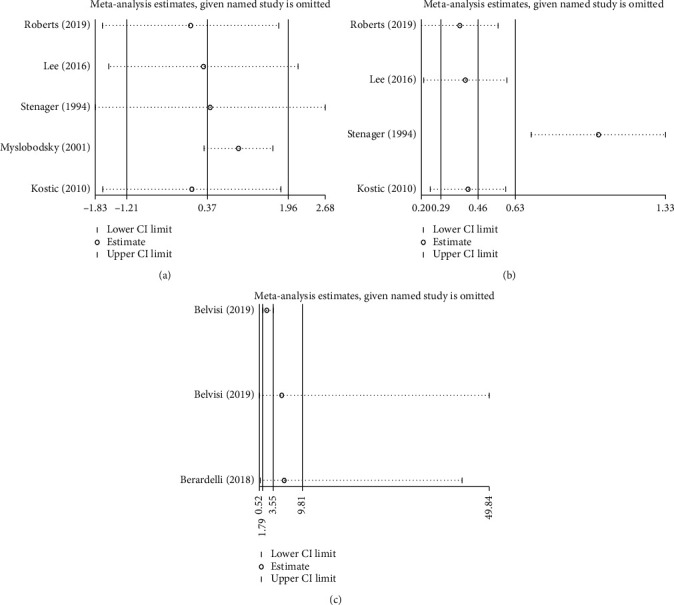
Sensitivity analysis of the included studies with significant heterogeneity comparing Parkinson's disease (PD) patients with general population or non-PD controls. (a) and (b) Among studies comparing PD patients with general population, Myslobodsky's study accounted for the heterogeneity. (c) Among studies comparing PD patients with non–central nervous system disease controls (healthy, psoriasis, or glaucoma), the data comparing PD patients with healthy controls accounted for nearly all heterogeneity.

Sensitivity analyses were performed as significant heterogeneity was shown among studies. For the 5 studies comparing the suicide risk of PD patients with general population, the pooled lnSMRs after excluding each study are shown in Figure 4. After removing Myslobodsky's study [6], which reported a much higher suicide rate of general population than other studies, the *I*^2^ decreased from 99.2% to 88.8% (*p* < 0.001). Excluding the other 4 studies did not significantly change the heterogeneity.

For the 2 articles comparing PD patients with healthy controls or patients with non–central nervous system diseases (psoriasis and glaucoma), after excluding the data comparing PD patients with healthy controls [15], the *I*^2^ decreased to 0 (*p*=0.693), while the pooled OR became 2.143 (95% CI: 1.297 to 3.539, *p*=0.003). This was supported by the fact that the data comparing PD patients with healthy controls presented a much higher suicide risk in PD patients (OR = 17.52, 95% CI: 4.04 to 75.91).

No significant heterogeneity existed among the 3 studies comparing PD patients who underwent DBS with PD patients who received only drug therapy, and the 2 studies comparing PD patients underwent DBS with general population.

### 3.5. Publication Bias

**Figure 5:**
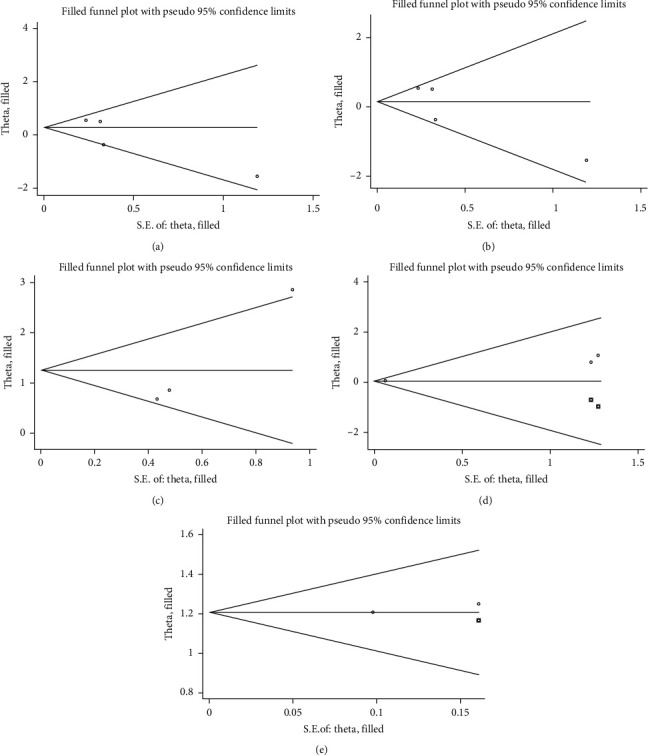
Funnel plots of publication bias analysis based on the trim-and-fill method. No significant publication bias was found in studies comparing (a) Parkinson's disease (PD) patients with general population. (b) After excluding Myslobodsky's study, there was still no significant publication bias. (c) Among studies comparing PD patients with non–central nervous system diseases (psoriasis and glaucoma) controls, significant publication bias was found. ((d) and (e) Significant publication bias was also found among studies comparing PD patients who underwent deep brain stimulation (DBS) with PD patients received only drug therapy, and among studies comparing PD patients who underwent DBS with general population.

Studies comparing the suicide risk in PD patients with general population showed no significant publication bias (*z* = 0.491, *p*=0.624). Similarly, no significant publication bias was found after excluding Myslobodsky's study [6] (Figure 5).

Studies comparing PD patients with healthy controls or patients with non–central nervous system diseases (psoriasis and glaucoma) presented a significant publication bias (*z* = 2.483, *p*=0.013).

Studies comparing PD patients who underwent DBS with PD patients who received only drug therapy presented a significant publication bias (*z* = 15.986, *p* < 0.001). Studies comparing PD patients who underwent DBS with general population presented also a significant publication bias (*z* = 16.290, *p* < 0.001).

## 4. Discussion

Initially, this meta-analysis identified a decreased risk of suicide and/or suicidal ideation in PD patients compared with general population. However, only Myslobodsky's study with the largest investigated population [6] among the 5 pooled studies reported a decreased risk of suicide in PD patients, while the other 4 reported an increased risk in PD patients. After excluding Myslobodsky's study [6], we found a significant increase in suicide and/or suicidal ideation in PD patients. Myslobodsky's study [6] was a case–control study performed in USA, which reported a really high risk of suicide in general population (0.8%), while the other 4 studies reported the risk of suicide and/or suicidal ideation in general population ranged from 0.012% to 0.37% [18, 20]. One possible explanation for the difference caused by Myslobodsky's study [6] could be attributed to its high suicide ratio in general population, which may possibly be explained by a relatively high suicide risk in USA [23, 24]. Besides, general cultural differences including European American ethnicity and male gender, comorbidities including bipolar disorders, depression, psychosis and alcohol use, and many treatments of PD may increase suicide risk in PD patients [25]. Interestingly, a study found that more hospital contacts, including full-time admission, outpatient visit, and emergency department contact, were positively correlated to higher suicide risk in PD patients [26], indicating a complicated effect of medical intervention, disease progression, and healthy conditions on suicide risk of PD patients. Therefore, it is important to take culture background and social factors [27] into consideration when analyzing suicide risk in PD patients. Importantly, Myslobodsky's study [6] presented many results contrary to common understanding of suicide risk. For example, it showed higher suicide risk in married PD patients rather than single ones, and it also showed that in all PD patients, suiciders had lower rate of somatic disorders than nonsuiciders, which even confused the authors themselves. Therefore, potential bias may exist in Myslobodsky's study [6], and we performed a second analysis after excluding Myslobodsky's study [6]. The other 4 studies were performed in England [18], South Korea [19], Denmark [17], and Serbia [20], and it is reasonable to speculate an increased risk of suicide and/or suicidal ideation in PD patients. Similarly, when combining the ORs of studies comparing suicide risk between PD patients and nongeneral population controls (healthy controls or patients with psoriasis or glaucoma) [15, 16], we also found a significantly increased risk of suicide in PD patients, supporting our speculation that PD may increase risk of suicide or suicidal ideation.

Notably, patients with chronic neurological disorders have been reported to present higher suicide incidence compared with non-neurological disorder patients, among which PD patients only showed a lower fully adjusted incidence rate ratio of suicide than patients with amyotrophic lateral sclerosis, Huntington disease, Guillain-Barré, multiple sclerosis, diseases of myoneural junction and muscles, and other (unclassified) brain disorders, ranking seventh together with epilepsy, encephalitis, and head injury in all investigated neurological disorders [26]. This report indicates that the increase of suicide risk in PD patients may be due to the nature of chronic neurological disorders rather than PD per se. Anyway, PD showed higher suicide risk compared with non-neurological disorder patients and many other neurological disorders, as the second-most common neurodegenerative disorder [1], the risk factors of suicide of PD need to be further researched.

In PD patients, non-motor symptoms including anxiety, depression, sleep disorder, cognitive impairment, and hyposmia are common [28], and these non-motor symptoms severely affect patients' quality of life, could lead to frailty and hopelessness, and finally may cause suicide [15]. Additionally, it has been reported that suicide risk in PD patients could be related to the frequency of motor symptoms, severity of non-motor symptoms, and patients' personal acceptance of PD-induced disability [16]. Among all these factors, depression is most recognized [29]. Depression was seen in 40% PD patients [30], and it was reported to increase the risk of suicide by 6.4 times [20]. Therefore, suicide risk in PD patients could at least partially be attributed to depression. Similarly, anxiety [31], sleep disorder [32], and other symptoms were also identified to contribute to risk of suicide. Quality of life was also significantly decreased in PD patients [33], which may bring mental problems including stigma, aloneness, and hopelessness [15], and all these could lead to an increased risk of suicide and/or suicidal ideation. It is important for clinical practitioners and caregivers to pay more attention to the mental status and risk of suicide in PD patients. Routine evaluation of the risk of suicide in PD patients should be encouraged in each stage of PD progression, and psychological intervention and social support should be started early when risk of suicide was identified. However, more studies are needed to further illustrate more risk factors of suicide in PD patients.

DBS is an effective therapy for PD, especially in patients with levodopa-related adverse effects such as dyskinesia [7]. DBS is generally safe in PD patients [8]; however, this meta-analysis found a trend of an increased risk of suicide and/or suicidal ideation in PD patients who underwent DBS compared with PD patients received only drug therapy, although there was no statistical significance. Due to the limited number of studies, it is difficult to further analyze the risk of suicide in PD patients who underwent DBS. Suicide and suicidal ideation or attempts have been observed in PD patients received DBS in STN or GPi [9, 10], even in patients whose motor symptoms were favorably relieved [10]. Risk factors associated with the increased risk of suicide in PD patients who underwent DBS were postoperative depression, being single, impulse control disorders, and younger age or onset at younger age [22]. The mechanism by which DBS induces suicide was speculated to involve serotonin [22]. Additionally, history of suicidal ideation/suicide attempts and psychotic symptoms, psychotropic medication use in both the preoperative and postoperative phases, family psychiatric history (notably of addiction, depression, and suicide), and verbalized suicidal ideation may be risk factors of suicide in PD patients who received DBS [21]. Besides, lack of satisfaction of DBS results and unrealistic expectations, poor supports, changes in identity or family/social interactions, adverse effects of DBS, and dopaminergic medication withdrawal (mainly present in the first months-years after surgery) are also related to higher risk of suicide after DBS [21]. These findings indicate that the risk of suicide in PD patients who underwent DBS is due to multiple factors and multidisciplinary assessment on patients' risk of suicide should be performed before DBS operation for their safety and good outcome. Close follow-up, support, and education on caregivers are also necessary. A scale for evaluating suicide risk in PD patients who preparing to receive DBS may be helpful for clinical practitioners.

Interestingly, we identified a significantly increased risk of suicide and/or suicidal ideation in PD patients who underwent DBS compared with general population, indicating a potential DBS-related increase in risk of suicide and/or suicidal ideation. Notably, PD patients have higher risk of suicide compared with general population [21, 22], and it is unclear whether this increased risk could be solely attributed to DBS per se or should be attributed to PD. Although many cases of DBS-related suicide were reported [34, 35], the specific DBS-increased risk of suicide and/or suicidal ideation has never been investigated. More studies are needed to further illustrate the association between DBS and risk of suicide. More importantly, attention should be paid to the psychological status and risk of suicide of PD patients who underwent DBS. Pre- and postoperative evaluations of patients' psychological status are necessary. Additionally, there were insufficient data for meta-analysis on the effect of different targeted locus of DBS on risk of suicide. The only one study reported similar risk of suicide between patients who received DBS in STN and GPi [10].

Notably, suicide risk has been noticed in patients who received either traditional neurosurgery or DBS. Epilepsy patients who underwent neurosurgery were reported to present a suicide incidence rate of 4593/year per 100000 population [36], with an SMR of 13.3 [37]. 12% brain tumor patients who underwent neurosurgery presented suicide ideation [38]. Meanwhile, a study found that 2 out of 16 dystonia patients who received DBS in GPi committed suicide [39], while 6 out of 140 patients who received DBS in motor nuclei of the thalamus, GPi or STN for treating PD, essential tremor, primary and secondary dystonia, and multiple sclerosis-associated tremor committed suicide, which presented a suicide incidence rate of 4.3% [34]. It is speculated that young age, male, more than one successive DBS, and previous history of depression or other psychiatric features are at higher risk of suicide [34]. On this aspect, the increased risk of suicide after DBS may be due to its damage to brain rather than DBS per se, especially considering that patients with brain trauma showed a suicide incidence of 4.6%, similar to DBS [40]. However, it has been reported that lowering stimulation frequency in a PD patient who received DBS in bilateral STN induced suicide attempt [41]. On this aspect, parameters of DBS may affect patients' suicide ideation or attempts, indicating that DBS per se may affect risk of suicide in patients. There are few studies that researched the mechanism by which DBS affects risk of suicide, and further study is needed.

Significant heterogeneity existed in studies comparing suicide risk of PD patients to general population. As discussed above, the heterogeneity could be attributed to Myslobodsky's study [6] because it reported a much higher suicide rate in general population and many findings contrary to common understanding of suicide risk. Heterogeneity was also identified in the 2 studies comparing PD patients to healthy controls or patients with non–central nervous system diseases (psoriasis and glaucoma). The sensitivity analysis showed that nearly all heterogeneity could be attributed to the study compared PD patients with healthy controls [15]. This result suggests that risk of suicide may be affected by disease conditions and diseases such as psoriasis and glaucoma may also affect risk of suicide in patients, although the risk of suicide in these patients was lower than PD patients. Significant publication bias was identified in studies comparing PD patients who underwent DBS with PD patients who received only drug therapy and studies comparing PD patients who underwent DBS with general population. This publication bias may be attributed to the limited number of existing studies. It is also possible that negative results are more likely not to be published.

Several limitations exist for this meta-analysis: (1) among all the included studies, some reported completed suicide and some reported completed suicide and suicidal ideation or attempts, which may affect the evaluation of risk of suicide; (2) diagnostic criteria among included studies differed; (3) only one study compared risk of PD patients with healthy controls, making it difficult to evaluate the specific risk of suicide induced by PD; (4) only risk of suicide in PD patients from limited countries were reported, considering the effect of social factors and culture background on suicide, more data are needed; (5) a limited number of studies were included in this meta-analysis; and (6) significant publication bias exists.

## 5. Conclusions

This meta-analysis found that PD patients have higher risk of suicide and/or suicidal ideation compared with controls, while DBS may increase the risk of suicide and/or suicidal ideation in PD patients. It is necessary for clinical practitioners and caregivers to pay attention to the mental status of PD patients. For patients planning to receive DBS treatment, psychological evaluation pre- and postoperation may also be important. Intervention and social support may be necessary for PD patients with psychological symptoms or PD patients at high risk of suicide. More investigations are needed to further illustrate the risk and risk factors of suicide in PD patients. The mechanisms of PD-related suicide also remain to be clarified.

## Data Availability

The data are available upon request to the corresponding author.

## Supplementary Materials

Supplementary Materials The supplementary materials consist of the following tables: 1. Table S1: characteristics of included studies comparing PD patients to non-PD controls. 2. Table S2: characteristics of included studies comparing PD patients underwent DBS to controls. 3. Table S3: quality assessment of cross-sectional studies. 4. Table S4: quality assessment of cohort studies. 5. Table S5: quality assessment of case-control studies. 6. Table S6: quality assessment of randomized control trial studies.
