# Supplementary material for: Parkinson's Disease-Related Risk of Suicide and Effect of Deep Brain Stimulation: Meta-Analysis
Source: Parkinsons Dis. 2020 Sep 27;2020:8091963. doi: 10.1155/2020/8091963 (PMC7537696; doi:10.1155/2020/8091963)
Supplement: Supplementary Materials — The supplementary materials consist of the following tables: 1. Table S1: characteristics of included studies comparing PD patients to non-PD controls. 2. Table S2: characteristics of included studies comparing PD patients underwent DBS to controls. 3. Table S3: quality assessment of cross-sectional studies. 4. Table S4: quality assessment of cohort studies. 5. Table S5: quality assessment of case-control studies. 6. Table S6: quality assessment of randomized control trial studies. [file 8091963.f1.docx]

**Table 1. Characteristics of included studies comparing PD patients to non-PD controls.**

| Study | Country | Study Design | PD patients (suicide/non-suicide) | Controls (suicide/non-suicide) | Type of Controls | Diagnostic criteria of PD | Ascertainment Method of Suicide and/or suicidal ideation | Risk estimates (95%CI) |
| --- | --- | --- | --- | --- | --- | --- | --- | --- |
| Belvisi D. 2019 | Italy | Cross-sectional | 31/69 | 2/78 | Healthy | EFNS/MDS-ES/ENS (2013) & MDS(2015) | The Columbia Suicide Severity Rating Scale (C-SSRS) | OR,17.5 (4.04-75.9) |
| Belvisi D. 2019 | Italy | Cross-sectional | 31/69 | 12/64 | Psoriasis | EFNS/MDS-ES/ENS (2013) & MDS(2015) | The Columbia Suicide Severity Rating Scale (C-SSRS) | OR,2.34 (1.33-5.06) |
| Berardelli I. 2018 | Italy | Cross-sectional | 31/69 | 17/74 | Glaucoma | EFNS/MDS-ES/ENS (2013) & MDS(2015) | C-SSRS, the Italian Perceived Disability Scale, Beck Hopelessness Inventory & TEMPS-A | OR,1.96 (0.99-3.85) |
| Roberts SE. 2017 | England | Cohort | 9/15768 | N/A | General population | ICD-10 | ICD-10 | SMR,5.6 (2.5-9.9) |
| Lee T. 2016 | South Korea | Cohort | 29/4333 | N/A | General population | Criteria for diagnosing PD, Ann. Neurol.32 (1992) S125eS127. | ICD-10 | SMR,1.99 (1.33-2.85) |
| Stenager EN.1994 | Denmark | Cross-sectional | 2/456 | N/A | General population | Clinical diagnosis | Deaths registration | SMR,1.24 (1.00-1.52) |
| Myslobodsky M. 2001 | USA | Case-control | 122/144241 | N/A | General population | N/A | ICD | SMR, 0.1 (0.084-0.12) |
| Kostić V. 2010 | Serbia | cohort | 2/100 | N/A | General population | British Brain Bank criteria | Recorded behavior | SMR,5.3  (2.1-12.7) |

CI, confidence interval; ICD, international classification of diseases; N/A, not applicable; OR, odds ratio; PD, Parkinson’s disease; SMR, standardized mortality ratio; USA, United States of America.

**Table 2. Characteristics of included studies comparing PD patients underwent DBS to controls.**

| Study | Country | Study Design | PD patients  Underwent DBS (suicide/non-suicide) | Controls (suicide/non-suicide) | Type of Controls | Targeted nucleus of DBS | Ascertainment Method of Suicide and/or suicidal ideation | Risk estimates (95%CI) |
| --- | --- | --- | --- | --- | --- | --- | --- | --- |
| Strutt AM. 2012 | UK | Cross-sectional | 1/16 | 0/22 | Non-surgical PD patients | STN | Mini Mental Status Exam, Beck Depression Inventory & State-Trait Anxiety Inventory | OR, 1.06 (0.94-1.2) |
| Weintraub D. 2013 | USA | RCT | 2/119 | 1/133 | PD patients with best medical therapy | STN or GPi | Suicide behavior that resulted in hospitalization or death | OR, 2.24 (0.2-24.97) |
| Lhommée E. 2018 | Europe | RCT | 2/15 | 1/22 | PD patients with medical therapy alone | STN | Beck Depression Inventory II & Starkstein Apathy Scale | OR, 2.93 (0.24-35.33) |
| Voon V. 2008 | World | Cohort | 24/5287 | N/A | General population | STN | Suicide risk factors questionnaire | SMR, 28.367 (15.43,53.43) |
| Giannini G. 2019 | France | Cohort | 4/530 | N/A | General population | STN | Beck Depression Inventory & behavior | SMR, 32.806 (11.31-95.186) |

CI, confidence interval; DBS, deep brain stimulation; GPi, globus pallidus internus; N/A, not applicable; OR, odds ratio; PD, Parkinson’s disease; RCT, randomized control trial; SMR, standardized mortality ratio; STN, subthalamic nucleus; UK, United Kingdom; USA, United States of America.

**Table 3. Quality assessment of cross-sectional studies.**

| Study | Study Design | Selection | | | | Comparability | Outcome | | Total score |
| --- | --- | --- | --- | --- | --- | --- | --- | --- | --- |
|  |  | Representative  Sample | Sample Size Adequate | Non-Respondents | Ascertainment of Exposure | Based on Design or Analysis | Assessment of Outcome | Statistical Test |  |
| Belvisi D 2019 | Cross-sectional | + | - | + | + | + + | + | + | 8 |
| Berardelli I 2018 | Cross-sectional | + | - | + | + | + + | + | + | 7 |
| Stenager EN 1994 | Cross-sectional | + | + | + | + | + | + | - | 7 |
| Strutt AM 2012 | Cross-sectional | + | - | + | + | + + | + | + | 7 |

**Table 4. Quality assessment of cohort studies.**

| Study | Study Design | Selection | | | |  | Comparability |  | Outcome | | | Total score |
| --- | --- | --- | --- | --- | --- | --- | --- | --- | --- | --- | --- | --- |
|  |  | Exposed Cohort Representative | Selection of Non-Exposed Cohort | Ascertainment of Exposure | Outcome not present at study start |  | Based on Design or Analysis |  | Assessment of Outcome | Timing of Follow-up | Adequate Follow-up |  |
| Roberts SE 2018 | Cohort | + | + | + | - |  | + - |  | + | - | + | 6 |
| Lee T 2016 | Cohort | + | + | + | + |  | + - |  | + | - | + | 7 |
| Kostić M 2010 | Cohort | + | + | + | + |  | + + |  | + | + | + | 9 |
| Giannini G 2019 | Cohort | + | + | + | - |  | + + |  | + | - | + | 7 |

**Table 5. Quality assessment of case control studies.**

| Study | Study Design | Selection | | | |  | Comparability |  | Exposure | | | Total score |
| --- | --- | --- | --- | --- | --- | --- | --- | --- | --- | --- | --- | --- |
|  |  | Case Definition Adequate | Cases Representative | Selection of Controls | Definition of Controls |  | Based on Design or Analysis |  | Ascertainment of Exposure | Same Method for Cases and Controls | Non-response Rate |  |
| Myslobodsky M 2001 | Case-control | + | + | + | + |  | + + |  | + | - | + | 8 |
| Voon V 2008 | Case-control | + | + | + | - |  | + + |  | + | + | - | 7 |

**Table 6. Quality assessment of randomized control trial studies.**

| Study | Study Design | Sequence Generation | Allocation  Concealment | Blinding | Incomplete Outcome Data | NO Selective Outcome Reporting | Other Sources of Bias | Total score |
| --- | --- | --- | --- | --- | --- | --- | --- | --- |
| Weintraub D 2013 | RCT | + | - | - | + | + | + | 4 |
| Lhommée E 2018 | RCT | + | + | + | + | + | + | 6 |

RCT, randomized control trial.
